# Contamination of Animal Feed with Undeclared Tetracyclines—Confirmatory Analysis by Liquid Chromatography–Mass Spectrometry after Microbiological Plate Test

**DOI:** 10.3390/molecules25092162

**Published:** 2020-05-05

**Authors:** Monika Przeniosło-Siwczyńska, Ewelina Patyra, Aleksandra Grelik, Maja Chyłek-Purchała, Beata Kozak, Krzysztof Kwiatek

**Affiliations:** Department of Hygiene of Animal Feedingstuffs, National Veterinary Research Institute, Partyzantow 57 Avenue, 24-100 Pulawy, Poland; ewelina.patyra@piwet.pulawy.pl (E.P.); aleksandra.grelik@piwet.pulawy.pl (A.G.); maja.purchala@piwet.pulawy.pl (M.C.-P.); beata.kozak@piwet.pulawy.pl (B.K.); kwiatekk@piwet.pulawy.pl (K.K.)

**Keywords:** antibiotics, tetracyclines, feed analysis, microbial screening assay, LC−MS

## Abstract

The presence of tetracycline (TC) antibiotics was determined in animal feed that had been previously screened with a microbiological plate test. Feed samples were screened by a microbiological plate test on a pH 6.0 culture medium seeded with *Bacillus cereus* ATCC 11778 able to pre-reveal the presence of tetracyclines. Subsequently, confirmation and quantification were performed using a validated HPLC method with mass spectrometric detection. In 2013–2018, 353 feed samples were analysed to detect antibacterial substances, of which 186 (52.7%) were suspected to contain tetracyclines. Forty-two out of 186 (22.6%) samples analysed by the chromatographic method contained undeclared tetracyclines, which were determined at concentrations from 0.3 to 49 mg kg^−1^. The most frequently identified contaminating tetracyclines were doxycycline and chlortetracycline.

## 1. Introduction

Tetracyclines (TCs) are antibiotics that exhibit activity against infections caused by both Gram-positive and Gram-negative bacteria as well as chlamydia, mycoplasmas, protozoan parasites and rickettsiae [1,2,3]. They are widely administered to intensive farming animals because of their broad spectrum of activity and cost-effectiveness [1,4]. Since their discovery in the late 1940s, TCs have been used widely for the treatment and prophylaxis of bacterial diseases and as feed additives to promote growth in farm animals [5,6,7]. The emergence of bacterial resistance against tetracyclines, and the emergence of bacterial resistance against antibacterial agents in general, has caused severe drawbacks in their use. Concerns were raised on the potential problems due to the use of human antibiotics for growth promotion in animal husbandry and the transference of resistance to humans [8,9,10]. The discussion concluded in 1974 with the elimination of TCs as feed additives along with other antibiotics used in humans [11].

Several properties make tetracyclines the most commonly used of all antibiotics: they are active against most common pathogens, show good oral absorption, exhibit low toxicity and cause only few allergic reactions, and they are relatively inexpensive [12]. This has led to an intensive use of TCs for the treatment and prophylactic control of bacterial infections not only in humans, but also in animals. Of the eight forms of commercially available tetracyclines, four are frequently used in animal husbandry: chlortetracycline (CTC), doxycycline (DC), oxytetracycline (OTC) and tetracycline (TC). According to data described by Grave et al. [13] on the consumption of veterinary antimicrobial agents intended for food-producing animals, in all European countries tetracyclines, penicillins and sulfonamides accounted for more than half (range 53–88%) of the total amount of antimicrobial agents sold by country, expressed as mg/PCU. This trend continues and, according to the ESVAC report [14] in 2017, the highest sales concerned tetracyclines, followed by penicillins and sulfonamides, accounting for 30.5%, 26.9% and 9.8%, respectively of the total sales of veterinary antimicrobial agents.

The usage of antimicrobials in farm animals is strictly regulated in the European Union (EU) to protect consumers, as the excessive and inappropriate use of antibiotics can lead to the emergence of bacteria resistant to antibiotics or the presence of antibacterial residues on food products of animal origin [15,16,17]. In the EU, antibacterial substances permitted as feed additives to help fatten livestock were prohibited for non-medicinal purposes in 2006. The control and analysis of antibacterial substances in feed for farm animals has become an important issue, as only authorized feeds can be medicated under specific conditions, as stated in Regulation (EU) 2019/4 [18]. The antibacterial substances, in addition to medicated feeds, can enter feed via different paths, for instance due to unintentional cross-contamination in the feed mills or by unauthorized use for prophylaxis or to fraudulently promote growth [19]. Cross-contamination between medicated and nonmedicated feed can occur with any type of drug added to the feed, particularly when the cleaning process between batches is inefficient [20]. The fact that antibiotics could be present as contaminants in feed without farmers’ knowledge implies that a withdrawal period will not be considered and, in addition to the development of antibiotic-resistant bacteria, antibiotic residues could remain in animal products (e.g., meat, eggs, milk) [21]. 

The effective and efficient control of the possible illegal use of undeclared antibiotics requires the availability of multi-screening and confirmation methods which can be implemented in an overall control strategy [22,23,24]. For the rapid detection of antibiotics in feed or food, microbiological assays are routinely used because they are easy to perform and inexpensive. However, the main disadvantage of these tests is their lack of specificity; that is, they often detect growth inhibition of a sensitive test strain, whether a substance with antimicrobial activity is present or not. The technique of choice for the confirmatory analysis of antimicrobial agents in feed is liquid chromatography coupled with mass spectrometry (MS). However, the analysis of animal feed has proved to be quite a challenge because of the high complexity and the variability of the matrix composition [25,26]. 

This study presents an antibiotics detection system based on a microbiological screening followed by a confirmation and quantification step consisting of a suitable chemical technique, such as HPLC with MS detection. This paper focuses on the analysis of the group of antibiotics approved by the EU for therapeutic use in pigs, poultry, cattle and sheep. The tetracyclines may be added to animal feed for treating the animals as a medicated feed. However, production practices in feed mills have been identified as the main source of feed contamination [27].

Because the use of antibiotics in feeds has been prohibited in the EU for non-medicinal purposes, monitoring for undeclared or illegal use of these substances is conducted within national programmes of feed control. This paper reports the investigation on the occurrence of tetracyclines in feed used in farm animals.

## 2. Results

Out of 353 feed samples, 186 were suspected for the presence of tetracyclines, because of the presence of an inhibition zone on the *B. cereus* ATCC 11778 plate. The LC−MS method was adopted for the confirmatory analysis of 186 feed samples found positive by the microbial assay. After LC−MS analysis, the presence of TCs was confirmed in 42 (22.6%) out of 186 suspect samples. Overall, the percentage of positive samples for TCs among all the analysed samples was 11.9%. The results are presented in Table 1. 

**Figure 1:**
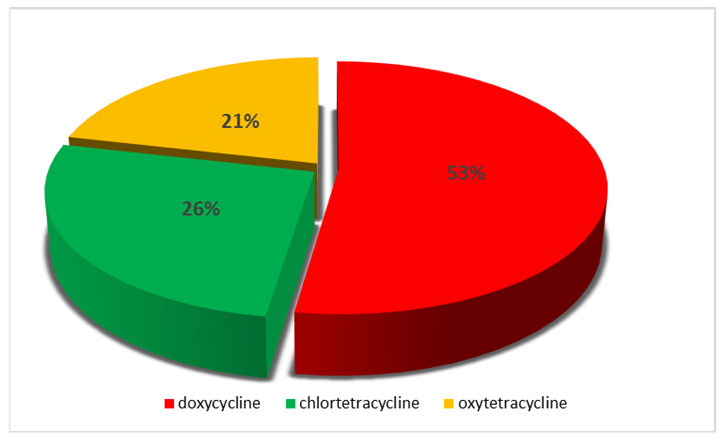
Percentage share of TCs revealed in the tested feed samples.

The most frequently identified antibiotic was doxycycline, followed by chlortetracycline (Figure 1). Doxycycline was found in 20 samples, and chlortetracycline in 11 samples. Moreover, oxytetracycline was detected in 9 samples. Furthermore, in 2 samples the presence of doxycycline in addition to tylosin was revealed. 

The compounds were found at concentrations from 0.3 to 49 mg kg^−1^. The results of the analytical determinations are presented in Table 2.

**Figure 2:**
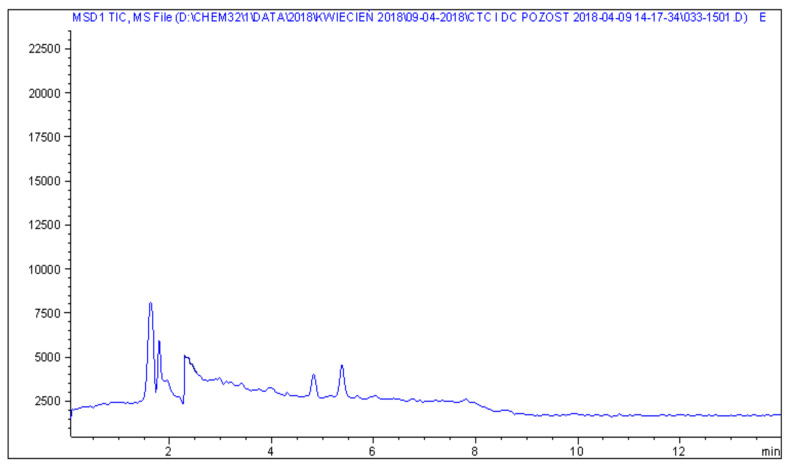
LC−MS chromatogram of a blank feed sample.

**Figure 3:**
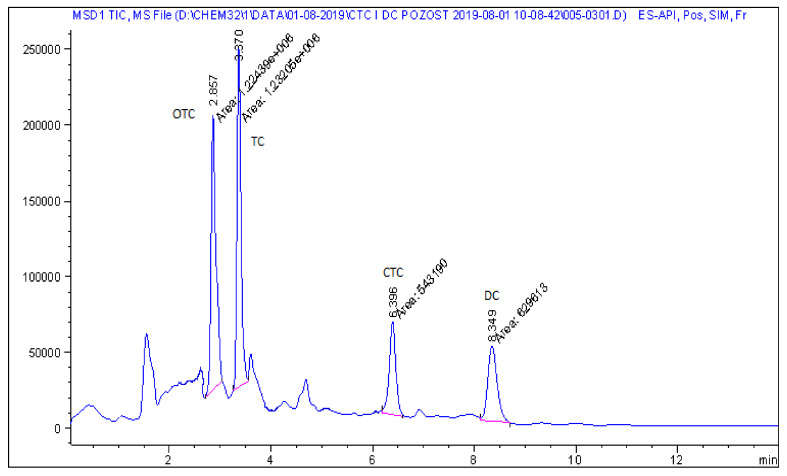
LC−MS chromatogram of a feed sample spiked with four tetracyclines at a concentration of 0.3 mg kg^−1^.

**Figure 4:**
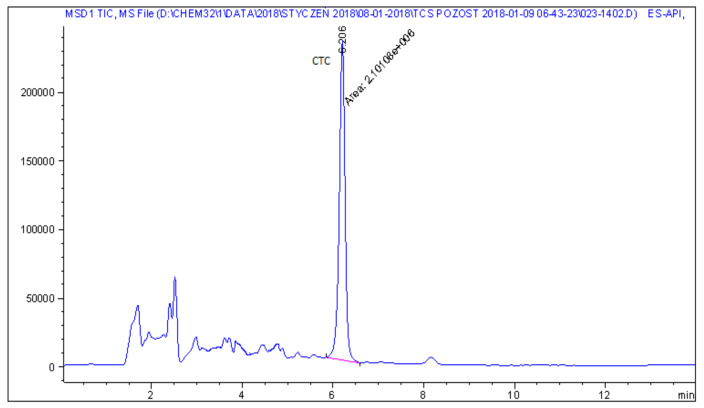
LC−MS chromatogram of real non-medicated feed sample with chlortetracycline at a concentration of 2.6 mg kg^−1^.

**Figure 5:**
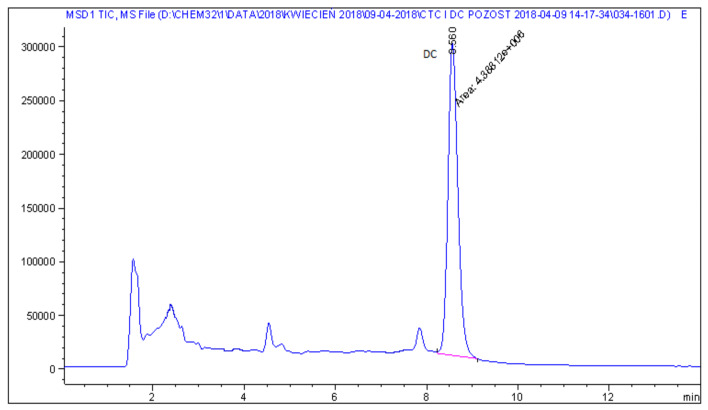
LC−MS chromatogram of real non-medicated feed sample with doxycycline at a concentration of 1.5 mg kg^−1^.

Representative chromatograms of blank feed sample, fortified feed sample and real feed samples with chlortetracycline and doxycycline, analysed by the described method, are shown in Figure 2, Figure 3, Figure 4 and Figure 5.

## 3. Discussion

Animal feeding has become an important economic activity and has a clear impact on food safety. Animal feeds, which are the main input into livestock production, not only require adequate quality from a nutritional point of view, but must also comply with legal limits regarding contaminants and be free from the unauthorized or unintentional presence of veterinary drugs. For this reason, the analysis and control of antibacterial agents in animal feed is a very important issue. Screening tests are often used to detect the presence of antimicrobials used in farm animals. The application of microbial assays for the screening of antibacterial substances in food or feed has been widely reported in the literature [15,22,23,24,28,29,30]. Microbiological assays are screening methods that are among the most commonly used techniques for the detection of the majority of antibiotic classes, especially if a large number of samples are to be analysed. On the other hand, screening methods can have acceptable false-positive result rates. Having false-positive results is tolerable for a screening test that must be very sensitive, but not very selective. Hence, due to the risk of false-positive samples, results from microbiological assays require confirmation. 

The eight-plate screening assay adopted in this study is based on the combination of pH conditions to favour or inhibit the antibiotic activity. Moreover, it is based on the sensitivity or resistance of the test microorganisms to various antibiotics. The combination of these factors enables a rough identification of antibiotics or antibiotic groups. In our method, eight plates used for a presumptive identification of six antibacterial families, including tetracyclines, proved to be a useful tool for the screening procedure. When all the plates are used, the pattern of inhibition can reduce unnecessary confirmatory analyses by indicating the antibacterial group most likely to be present. One of the eight plates is designed for the detection of tetracyclines owing to its low pH and a sensitive bacterial strain. Originally, the tetracycline family was the only one detected on the plate inoculated with *B. cereus* ATCC 11778. 

To confirm the results obtained with the microbiological assay and to supply quantitative data, an HPLC analysis with mass-spectrometric detection was performed on the suspected and positive samples. Confirmation with HPLC or a similar technique is always necessary to identify and quantify analytes after a screening test. Due to the risk of false-positive samples, results from microbiological assays require confirmation by a confirmatory method, allowing for the selective, sensitive and accurate detection and quantification of antibiotics for effective surveillance. 

Animal feed is a very complex matrix. Different animal species require different nutritive doses to get an equilibrated diet and be able to develop their specific productive functions. Poultry feeds in particular are characterized by a complex composition. In poultry, mainly commercial feeds are used; this lies in contrast to pigs, which are fed mostly production feeds. In general, feed composition may include, for example, amino acids, minerals, vitamins and salts, but also additives like organic acids, herbal mixtures or coccidiostats, or any other non-specific antibacterial substances (inhibitors) which induce matrix interference that may cause false-positive results in microbial assays. The lack of confirmed samples among poultry feeds as well as in concentrates/premixes shows that the presence of these interfering components can influence the occurrence of false-positive results. Nevertheless, microbiological methods play a crucial role in the effective official feed control and analyses.

The results showed that tetracycline antibiotics were identified in 42 (11.9%) of the analysed samples. Doxycycline and chlortetracycline were found in a total of 31 (73.8%) samples. The levels of antibiotics ranged from 0.3 to 5 mg kg^−1^. Low levels of contamination suggest cross-contamination during manufacturing in the feed mill or on the farm, although in Canada pigs can receive feed supplemented with subtherapeutic doses of chlortetracycline, at 5.5 mg kg^−1^ [31]. However, the presence of oxytetracycline may be surprising, because in Poland there are no approved medicated premixes with this antibiotic to manufacture medicated feed; thus, it may suggest the illegal use of oxytetracycline on farms. This could be confirmed by quantified relatively high concentrations ranging from 0.9 to 49 mg kg^−1^. Moreover, two feed samples contained doxycycline together with tylosin at concentrations of 7.9 and 10.9 mg kg^−1^ and 3.7 and 0.4 mg kg^−1^, respectively, indicating either the illegal use of the antibiotics or cross-contamination. Cross-contamination during the production process in feed mills can be an important reason for the contamination of feed with undeclared drugs [27,30]. In the survey described by Lynas et al., the most frequently detected contaminant was chlortetracycline which was found in 15.2% of all analysed feedingstuffs. Furthermore, a study conducted in the Netherlands confirmed that nonmedicated feed batches were contaminated with antibiotic residues such as tetracyclines, penicillins and sulfonamides [20]. Out of 140 analysed samples, 87% of the samples contained detectable amounts of antibiotics, with the concentrations ranging from 0.1 to 154 mg kg^−1^. However, our own research revealed the unconscious use of chlortetracycline-contaminated feed on few pig farms. The feed was manufactured by a medicated-feed-producing mill. The concentrations of contaminating chlortetracycline were between <0.3 and 2.7 mg kg^−1^.

Usually, cross-contamination results in subtherapeutic concentrations in feeds which are unlikely to equal those prescribed for medication. In spite of this, they can sometimes be sufficient to cause violative residues in meat or meat products. Thus, the antibacterial substances that enter the feed via different paths can pass through the food chain to the consumers, causing possible allergic reactions, toxic effects and bacterial resistance problems. Feed contaminated with pharmacologically active substances can lead to the sale of food of animal origin containing drug residues, for which the zero level applies, or at concentrations greater than the authorized maximum residue limits MRLs. For example, Segato et al. [32] conducted studies in order to obtain information on the transfer of doxycycline to chicken tissues when feed contaminated by this antibiotic at the level of 4 mg kg^−1^ was administered to poultry. Under the adopted experimental conditions, the doxycycline concentrations in each tissue (muscle, liver, kidney) reached average levels lower than the corresponding MRL, but the analyte was present. This study showed the role played by feed; in the absence of the withdrawal period, contaminating drugs can pose a risk to the consumers due to the occurrence of residues.

Animal feeds must have the required quality, especially in the context of food chain safety. This is very significant in the event that antimicrobials might provoke allergies and contribute to the development of resistant bacterial strains if they reach the food chain. An exposure to low concentrations of antimicrobials may induce resistance in the normal gut bacteria of food animals, which could then be transferred to pathogenic organisms. Tetracyclines are one of the most commonly used groups of antibiotics in animal production. At the same time, they are categorized as highly important antimicrobials in human medicine [33]. The evidence for a link between non-human sources (animals, food, or the environment) and the potential to cause human disease is greatest for certain bacteria (e.g., non-typhoidal *Salmonella*, *Campylobacter* spp., *Escherichia coli*). Monitoring of antimicrobial resistance in commensal *Escherichia coli* isolated from slaughtered broilers, laying hens, turkeys, swine and cattle carried out in Poland between 2009–2012 revealed that antimicrobial resistance reached the highest values for tetracycline (43.3%), followed by ampicillin (42.3%) and ciprofloxacin (39.0%) [34]. Researchers consider that antibiotic use in the life cycle of food animals makes it more likely that zoonotic foodborne bacteria such as *Salmonella* and *Campylobacter* harboured by the animals will be resistant to common antibiotics—especially if they are used at subtherapeutic concentrations. An increase in the prevalence of tetracycline-resistant *Salmonella* or *Campylobacter* strains may have implications for the antibiotic treatment of salmonellosis or campylobacteriosis cases in humans. However, studies carried out by Holman [31] indicated that chlortetracycline appears to have no effect on the measurable chlortetracycline resistance when given at a concentration of 5.5 mg kg^−1^ feed.

## 4. Materials and Methods

### 4.1. Sample Collection

Feed samples were provided to the laboratory by inspectors, feed producers and animal holders and were collected from all over Poland. The samples were taken as a part of the national feed monitoring programme and commercial research. During 2013–2018, a total of 353 samples of feedingstuffs were analysed. This number was comprised of 52 samples of cattle feed, 138 samples of pig feed, 114 samples of poultry feed, 21 samples of concentrates/premixes and 28 samples of feed materials. The types of analysed samples are present in Table 1. 

### 4.2. Microbiological Plate Test

In the first stage, the tetracycline antibiotics were detected by a microbiological 8-plate screening method for detection of antibacterial substances in animal feedingstuffs, which was developed and validated in our laboratory [16,35]. Combination of agar medium, pH, and test microorganisms was optimised to yield a suitable sensitivity and optimal conditions for the detection of a single group of antibiotics. For tetracycline antibiotics, agar medium pH 6.0 and test microorganism *Bacillus cereus* ATCC 11778 were used. The limit of detection of this method for tetracyclines was 0.3 mg kg^−1^. To perform a sample assay, sterile petri dishes (diameter, 120 mm) were filled with 20 mL of inoculated medium and left to harden at room temperature on a horizontal surface. When the agar was solidified, two wells were punched with a cork borer of 11 mm diameter on the plate. The plates were subjected to a quality control when unknown samples were analysed. Two antimicrobial disks (tetracycline 30 µg, Oxoid) were placed on the control petri dish to confirm standardization. 

**Figure 6:**
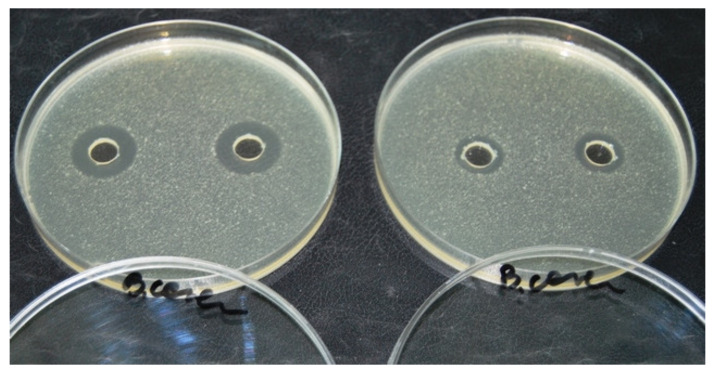
The presence of growth inhibition zones on *B. cereus* ATCC 11778 plates.

A ten-gram portion of the sample was then weighed into 200 mL Erlenmeyer flask. The samples were extracted with 50 mL of methanol/phosphate buffer (pH 8.0) mixture (1:1, *v*/*v*). The extraction time was 30 min. After the extraction, the samples were centrifuged for 10 min at 3000 × g and 100 µL of the supernatants was pipetted into wells on the agar surface on the plates. Test plates were incubated overnight at 30 °C, and then inspected for inhibition zones around the wells. Results of the microbiological assay were obtained by analysing the effect of diffusion on the agar medium inoculated with bacterial strain *Bacillus cereus* ATCC 11778. The presence of antibacterial substances was shown by the formation of growth inhibition zones around the punch hole after overnight incubation, as shown on Figure 6. The diameter of the zones was measured and a positive result was defined by an inhibition zone greater than 14 mm. The samples showing growth inhibition zones on the test plate designed for TCs were suspicious of TCs and analysed and confirmed by liquid chromatography mass spectrometry. 

### 4.3. Liquid Chromatography–Mass Spectrometry Analysis

An analytical method for simultaneous determination of oxytetracycline, tetracycline, chlortetracycline and doxycycline using liquid chromatography–mass spectrometry was developed and validated as described by Patyra and Kwiatek [36]. 

The HPLC–MS consisted of HPLC Agilent 1200 and MS Agilent 6140 (Agilent Technologies, Santa Clara, CA, USA) controlled by the software ChemStation (Agilent Technologies, Santa Clara, CA, USA). The chromatographic analyses were performer by injecting 15 µL of extract into a Kinetex C18 column (100 × 4.6 mm i.d., 2.6 µm) (Phenomenex, Torrance, CA, USA). Mobile phases A and B were mixed on a gradient mode with a flow rate of 0.5 ml min^−1^. The column temperature was set at 20 °C. Analytes were detected in positive electrospray mode (ESI+). The monitored precursor ions for OTC, TC, CTC and DC were 461, 445, 479 and 445 *m/z*, respectively. 

#### 4.3.1. Chemicals, Reagents and Stock Solutions

Oxytetracycline, tetracycline, chlortetracycline, doxycycline, formic acid and ethylenediaminetetraacetic acid disodium salt (Na_2_EDTA) were purchased from Sigma-Aldrich (St. Louis, MO, USA). Acetonitrile and methanol (HPLC grade) were purchased from J.T. Baker (Deventer, the Netherlands). Sodium hydroxide and sodium hydrogen phosphate were obtained from Avantor Performance Materials (Gliwice, Poland) and citric acid from Acros Organics (Geel, Belgium). All reagents used were of analytical grade and analytically pure. Water was deionised (>18 MΩ cm^−1^) by the Milli-Q water purification system (Bedford, MA, USA).

The McIlvaine-Na_2_EDTA buffer was prepared by dissolving 11.406 g Na_2_EDTA in 115.65 mL 0.2 M phosphate buffer and 184.65 mL 0.1 M citric acid. The pH was adjusted to 4.0 by adding 0.1 M citric acid or 0.2 M phosphate buffer. Mobile phase A consisted of Milli-Q water acidified to 0.1% with formic acid, and mobile phase B consisted of acetonitrile acidified to 0.1% with formic acid. 

To prepare the individual stock solutions of tetracyclines, 10 mg of tetracycline was dissolved in 10 mL of methanol and stored at −20 °C for up to six months. A working standard solution of tetracyclines was prepared by diluting the stock solution of each tetracycline to yield a final concentration of 100 µg mL^−1^ (the purity was consider when calculating appropriate concentrations) and stored at −20 °C for up to six months. 

#### 4.3.2. Sample Preparation and Clean-Up

Firstly, matrix-matched calibration curves were prepared. Different volumes of the tetracyclines working solutions were added to 5 g blank feed samples. Concentrations of tetracyclines in the matrix-matched feed samples were 0, 0.3, 0.5, 1.0, 2.5 and 5.0 mg kg^−1^.

To extract the tetracyclines from the feed samples, 5 g of grounded feed and 25 mL of McIlvaine-Na_2_EDTA buffer were added to a 50 ml polypropylene tube. After shaking for 45 min on a horizontal shaker, the sample was centrifuged at 4000× *g*. Then, 5 mL of the supernatant was loaded onto a Strata-X-CW SPE cartridge (300 mg, 3 mL) that had been preconditioned with 3 mL of methanol and 3 mL of water. The cartridges were then rinsed with 6 ml of water, 6 mL of methanol and 3 mL of acetonitrile with 2% formic acid. The analytes were eluted with 3 mL of methanol with 2% formic acid. The eluate was evaporated to dryness under a nitrogen stream and the final residue was dissolved in 1 mL of 0.1% formic acid in water. Then, the extract was put into 2-mL autosampler vials and 15 µL were injected into the HPLC−MS system.

## 5. Conclusions

In conclusion, animal feed can be screened for the presence of antibacterial substances, including tetracyclines with a microbiological inhibition test, using a solid medium at pH 6.0 and *B. cereus* as a test organism. Confirmation with the HPLC technique is always necessary to gain information about the substance identification and quantification. Our study shows that tetracycline antibiotics may occur as contaminating agents in animal feed. A major implication of contamination of feed with undeclared antimicrobial agents is, firstly, the contribution to the development of bacteria resistant to drugs used to treat infections, and secondly, the risk concerning residues of antibiotics in edible tissues and products that can produce allergic or toxic reactions in consumers, particularly if it concerns antibiotics that are absorbed in the digestive tract [37,38].

## Figures and Tables

**Table 1:** The number of samples contaminated with tetracyclines (TCs).

Type of Sample	Number of Analysed Samples	Number of Samples Suspected of TCs	Number of Samples with TCs Confirmed
Cattle feed	52	9	1
Pig feed	138	69	38
Poultry feed	114	79	0
Concentrates/premixes	21	19	0
Feed Materials	28	10	3
Total	353	186 (52.7%)	42 (11.9%)

**Table 2:** The analytes and their concentrations detected in samples with an indication of the type of feed.

Sample No.	Type of Feed	Analyte	Concentration (mg kg^−1^)
1	Cattle feed	CTC	0.3
2	Porker feed	CTC	0.35
3	Sow feed	DC	0.36
4	Porker feed	CTC	0.85
5	Piglet feed	DC	4.1
6	Pig feed	DC	1.4
7	Porker feed	DC	1.4
8	Sow feed	DC	5.0
9	Pig feed	OTC	4.7
10	Pig feed	OTC	>5.0 (49) *
11	Pig feed	OTC	1.8
12	Pig feed	DC	4.6
13	Pig feed	OTC	>5.0 (8.1)
14	Pig feed	OTC	0.9
15	Sow feed	DC	1.2
16	Porker feed	DC	<0.3
17	Porker feed	DC	<0.3
18	Porker feed	OTC	2.3
19	Porker feed	DC	0.9
20	Pig feed	DC	4
21	Pig feed	CTC	1.2
22	Pig feed	CTC	1.1
23	Pig feed	CTC	<0.3
24	Pig feed	CTC	2.6
25	Pig feed	CTC	<0.3
26	Pig feed	CTC	2.7
27	Pig feed	CTC	2.6
28	Porker feed	OTC	2.8
29	Piglet feed	DC	0.57
30	Piglet feed	DC	1.5
31	Pig feed	DC	0.47
32	Sow feed	OTC	>5.0 (5.5)
33	Pig feed	DC	0.57
34	Porker feed	CTC	0.33
35	Piglet feed	DC	0.6
36	Porker feed	DC	2.4
37	Pig feed	OTC	>5.0 (5.7)
38	Porker feed	DC (+tylosin)	>5.0 (7.9) (+10.9)
39	Piglet feed	DC (+tylosin)	3.7 (+0.4)
40	Feed material	DC	0.6
41	Feed material	DC	0.5
42	Feed material	DC	0.6

* The determined concentration was above the working range of the method. Approximate content is indicated in the brackets (calculated by means of a calibration curve). CTC: chlortetracycline; DC: doxycycline; OTC: oxytetracycline.
